# Anticoagulant Activity of *Naja nigricollis* Venom Is Mediated by Phospholipase A2 Toxins and Inhibited by Varespladib

**DOI:** 10.3390/toxins13050302

**Published:** 2021-04-23

**Authors:** Taline D. Kazandjian, Arif Arrahman, Kristina B. M. Still, Govert W. Somsen, Freek J. Vonk, Nicholas R. Casewell, Mark C. Wilkinson, Jeroen Kool

**Affiliations:** 1 Centre for Snakebite Research and Interventions, Liverpool School of Tropical Medicine, Pembroke Place, Liverpool L3 5QA, UK; Taline.Kazandjian@lstmed.ac.uk (T.D.K.); Nicholas.Casewell@lstmed.ac.uk (N.R.C.); 2 Department of Chemistry and Pharmaceutical Sciences, Division of Bioanalytical Chemistry, Faculty of Sciences, Amsterdam Institute of Molecular and Life Sciences (AIMMS), Vrije Universiteit Amsterdam, De Boelelaan 1083, 1081HV Amsterdam, The Netherlands; a.arahman@vu.nl (A.A.); k.b.m.still@vu.nl (K.B.M.S.); g.w.somsen@vu.nl (G.W.S.); 3 Centre for Analytical Sciences Amsterdam (CASA), 1012WX Amsterdam, The Netherlands; 4 Faculty of Pharmacy, Kampus Baru UI, Universitas Indonesia, Depok 16424, Indonesia; 5 Naturalis Biodiversity Center, Darwinweg 2, 2333CR Leiden, The Netherlands; freek.vonk@naturalis.nl

**Keywords:** snakebite, *Naja nigricollis*, coagulopathy, varespladib, marimastat, nanofractionation, mass spectrometry

## Abstract

Bites from elapid snakes typically result in neurotoxic symptoms in snakebite victims. Neurotoxins are, therefore, often the focus of research relating to understanding the pathogenesis of elapid bites. However, recent evidence suggests that some elapid snake venoms contain anticoagulant toxins which may help neurotoxic components spread more rapidly. This study examines the effects of venom from the West African black-necked spitting cobra (*Naja nigricollis*) on blood coagulation and identifies potential coagulopathic toxins. An integrated RPLC-MS methodology, coupled with nanofractionation, was first used to separate venom components, followed by MS, proteomics and coagulopathic bioassays. Coagulation assays were performed on both crude and nanofractionated *N. nigricollis* venom toxins as well as PLA_2_s and 3FTx purified from the venom. Assays were then repeated with the addition of either the phospholipase A_2_ inhibitor varespladib or the snake venom metalloproteinase inhibitor marimastat to assess whether either toxin inhibitor is capable of neutralizing coagulopathic venom activity. Subsequent proteomic analysis was performed on nanofractionated bioactive venom toxins using tryptic digestion followed by nanoLC-MS/MS measurements, which were then identified using Swiss-Prot and species-specific database searches. Varespladib, but not marimastat, was found to significantly reduce the anticoagulant activity of *N. nigricollis* venom and MS and proteomics analyses confirmed that the anticoagulant venom components mostly consisted of PLA_2_ proteins. We, therefore, conclude that PLA_2_s are the most likely candidates responsible for anticoagulant effects stimulated by *N. nigricollis* venom.

## 1. Introduction

Snakebite has been defined by the World Health Organization (WHO) as a neglected tropical disease since June 2017. Though estimates of global envenoming rates fluctuate dramatically, it is thought that between 421,000 [1] and 2.7 million [2] people per year are bitten by venomous snakes, resulting in an estimated 81,000–138,000 deaths annually. Reliable estimates of snakebite cases are challenging to obtain due to data being reliant on geographically-focused surveys and hospital statistics [3], while issues arise from the underreporting of snakebite incidences from both individuals and hospitals [4]. The most recent efforts at mapping snakebite vulnerability place 92.66 million people as living in areas vulnerable to snakebite, with the most vulnerable of these areas located in sub-Saharan Africa [5].

Elapids (Elapidae: including cobras, mambas, and related species) are a venomous, front-fanged family of snakes responsible for many snakebite cases in Africa. Bites from these snakes often result in severe neurotoxic pathology, resulting in initial symptoms of ptosis before progressing (in the worst of cases) to total respiratory paralysis and death [6,7]. Cobras (*Naja* spp.) are a group of elapid snakes that display defensive hooding behaviour when threatened, and bites from many species inflict neurotoxic symptoms typical of other elapid venoms [8,9,10]. However, African spitting cobras (subgenus *Afronaja*), which can project their venom as a defensive spray, more typically inflict bites that result in cytotoxic effects, such as swelling and tissue necrosis, as well as haemostatic disturbances, such as abnormal blood clotting [7,11,12,13]. The black-necked spitting cobra, *Naja nigricollis*, is a medically-important species that inhabits a wide range across the central belt of Africa [14], and is thought to be responsible for the majority of elapid bites in many of these areas [15,16,17]. Bites from this species have been shown to result in local swelling and tissue necrosis, bleeding, and blistering [11,13]. In vivo studies show that, when injected intradermally, the venom causes oedema, blistering and necrotic lesions in a mouse model [18], and exhibits strong cytotoxic effects on the myogenic cell line C2C12 [19].

Anticoagulant toxins may play an important role in certain elapid envenomings by acting as a spreading factor for other components such as neurotoxins [20]. These toxins cause anticoagulant effects via several mechanisms: through the inhibition of factor X or thrombin, the lysis of fibrin, the activation of plasminogen and/or the inhibition of platelet aggregation [21,22]. Several toxin families found in the venoms of cobras are thought to exert functional effects consistent with causing anticoagulation. Phospholipases A_2_ (PLA_2_s) from *N. naja* and *N. haje* venom have been previously demonstrated to inhibit platelet aggregation [23,24] and thrombin [25], respectively. Cytotoxic three-finger toxins (3FTxs) from *N. atra* and *N. kaouthia* venoms have been demonstrated to lyse erythrocytes [26], while the cytotoxic 3FTx complex hemextin AB identified from the venom of the closely related species *Hemachatus haemachatus* has been shown to inhibit the activity of the coagulation component factor VIIa [27]. The apparent synergy between PLA_2_s and 3FTxs in *N. mossambica* and *H. haemachatus* venom has been found to increase the potency of haemolytic venom activity [28], and perhaps cytolysis in general [29]. Some snake venom serine proteases (SVSPs), though not typically a major constituent of elapid venoms [30], can contribute to coagulant activity through the activation of clotting factors V/Va and VIII/VIIIa, thereby acting in a procoagulant manner [31]. Other SVSPs are anticoagulant via their cleavage of fibrinogen, thus depleting the availability for fibrin formation caused by this activity [32]. Finally, snake venom metalloproteinases (SVMPs) from *N. naja* venom have been found to cause delays in clotting time through the hydrolysis of haemoglobin [33]. However, in combination, 3FTxs, PLA_2_s and SVMPs typically account for >95% of the total toxin content of the majority of cobra venoms [29], and thus identifying the key toxins responsible for anticoagulant venom effects remains challenging. Nonetheless, a recent study showed that the PLA_2_ inhibitor varespladib was capable of effectively neutralising the anticoagulant activity of many Afro-Asian elapids, including *N. nigricollis* [34], suggesting that this toxin family may be of greatest importance.

The venom of *N. nigricollis*, like many elapid snakes [30], is predominantly composed of 3FTxs and PLA_2_s [29,35]. Crude *N. nigricollis* venom shows the ability to cleave fibrinogen [36] and inhibit platelet aggregation [37], but the primary toxin thought to be responsible for anticoagulation appears to be a multifunctional PLA_2_ named “CM-IV”. Historical literature suggests that CM-IV causes anticoagulant effects via the inhibition of the extrinsic coagulation cascade and the tenase and prothrombinase complexes [38,39,40], and via non-enzymatic binding to clotting factor Xa [41],. In addition, and likely, secondarily, four different 3FTxs found in *N. nigricollis* venom have been demonstrated to lyse erythrocytes [42].

**Figure 1:**
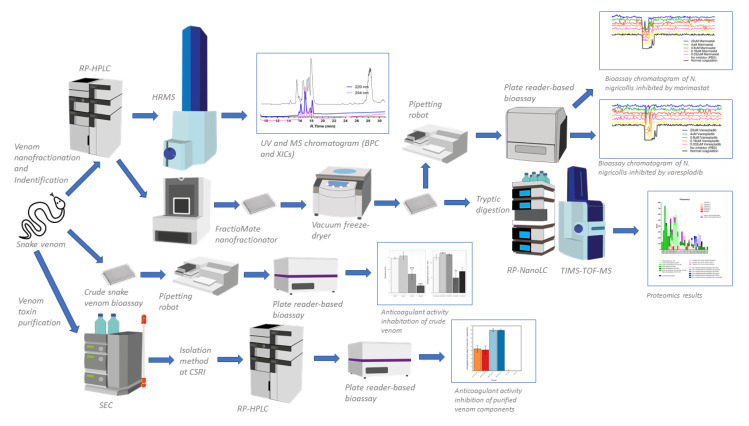
A schematic overview of the complete analytical and biochemical workflow. There are three main experiments that run simultaneously in this study: (i) crude snake venom plate reader-based bioassay (resulting inhibition anticoagulant activity by small molecule), (ii) venom separation (using reversed phase high performance liquid chromatography mass spectrometry (RP-HPLC-MS)) coupled with nanofractionation, resulting UV and MS data), bioassaying anticoagulant activity on 384-well plates (resulting bioassay chromatogram), and identification using proteomics (resulting protein hits), (iii) purification of snake venom using standard chromatography and plate reader-based bioassay (resulting anticoagulant activity). Trapped ion mobility spectrometry time of flight mass spectrometry (TIMS-TOF-MS). TIMS was not used in this study. High resolution mass spectrometry (HRMS). Reversed phase nanoflow liquid chromatography (RP-nanoLC). Size exclusion chromatography (SEC).

This study aims to reinvestigate the anticoagulant activity of venom from the medically-important black-necked spitting cobra (*Naja nigricollis*) and identify the toxins responsible for perturbing blood clotting. Crude venom activity was first analysed through a coagulation assay on bovine plasma [43], demonstrating a potent anticoagulant effect. A two-fold approach was then used to identify coagulopathic toxins: i) nanofractionation of *N. nigricollis* venom was performed followed by MS and proteomics-based identification of anticoagulant venom toxins detected in the bioassay and ii) venom was separated by three steps of chromatography into its main constituent parts, PLA_2_s and 3FTxs, and these toxins were then assessed for anticoagulant activity, A schematic overview of the complete analytical and biochemical workflow including workflow explanation is provided in Figure 1. Ultimately, the combination of these experimental approaches convincingly demonstrated that PLA_2_s are responsible for disruptions in coagulant activity caused by *N. nigricollis* venom and that this activity is effectively inhibited by varespladib.

## 2. Results

### 2.1. Anticoagulant Effects of Crude N. nigricollis Venom and Quantification of Inhibition by Small Molecule Toxin Inhibitors

**Figure 2:**
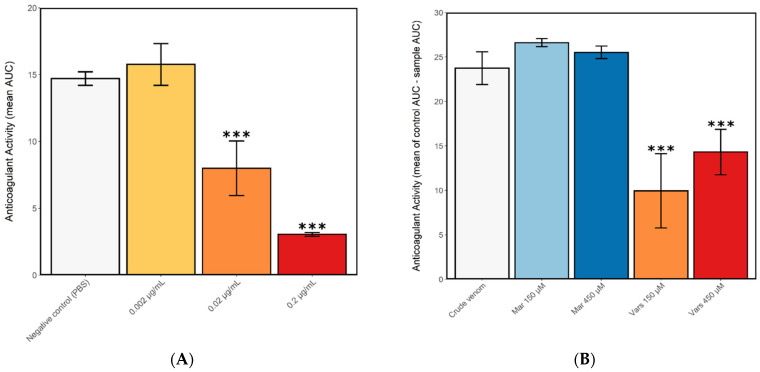
Crude *N. nigricollis* venom exerts anticoagulant effects, even at small doses, and this activity is inhibited by the small molecule phospholipase A_2_ inhibitor varespladib. The plasma clotting ability of (**A**) crude *N. nigricollis* venom at three different concentrations and (**B**) 2 µg/mL venom in the presence of the inhibitors varespladib (150 and 450 µM) or marimastat (150 and 450 µM). Anticoagulant activity was measured as the mean area under the curve (AUC) of light absorption at 595 nm, from 0 to 100 min assay time (displayed in a) and the mean control area under the curve AUC minus the mean AUC of each sample (displayed in b). Mar-marimastat, Vars-varespladib. Asterisks indicate statistically significant values from the crude venom sample (*** *p* < 0.005). Error bars represent the standard error of the mean (SEM).

To analyse the effects of *N. nigricollis* venom on blood coagulation, crude venom was applied to citrated bovine plasma in a 384-well plate bioassay. The presence of crude venom at assay concentrations of 0.2 µg/mL and 0.02 µg/mL, but not 0.002 µg/mL, was found to significantly decrease coagulant activity as compared to control wells containing PBS only (one-way ANOVA with multiple comparisons: *F* = 33.1, *R2* = 0.67, *p* < 0.0001), confirming that *N. nigricollis* venom has a potent anticoagulant effect even at low doses (Figure 2a). Next we tested the potential of toxin family-specific small molecule inhibitors to inhibit the anticoagulant effect of *N. nigricollis* venom, and demonstrated that a 150 µM concentration of the PLA_2_ inhibitor varespladib caused a significant decrease in anticoagulant activity compared to crude venom (one-way ANOVA with multiple comparisons: *F* = 8.67, *R2* = 0.73, *p* = 0.01), but not a 450 µM concentration, though this did approach the significance threshold (*F* = 8.67, *R2* = 0.73, *p* = 0.08) (Figure 2b). Contrastingly, the SVMP inhibitor marimastat showed no inhibition of anticoagulant venom activity at either of the tested concentrations (*F* = 8.67, *R2* = 0.73, *p* = 0.84; *p* = 0.97, for 150 µM and 450 µM, respectively) (Figure 2b), indicating that SVMPs are not likely to be involved in the anticoagulant activity of *N. nigricollis* venom.

### 2.2. Identification of Anticoagulant Venom Toxins Via Nanofractionation, Bioactivity Testing and Proteomics

To further explore the toxins responsible for anticoagulant venom effects, we next applied an approach consisting of high-resolution LC fractionation (i.e., nanofractionation) of venom (50 μL per injection at various concentrations) into a 384-well plate followed by the use of the plasma coagulation assay. Venom was separated using HPLC, followed by a post-column split in a 1:9 ratio, of which the smaller portion went to UV and MS detection, while the majority went to the nanofractionation module, which enabled high-resolution column eluate collection in serpentine-wise fashion onto 384-well plates. After performing the coagulation bioassay on the plate with fractionated toxins, so-called bioassay chromatograms were constructed for which the coagulation bioassay signal was plotted (y-axis) versus retention time of fractionation (x-axis).

**Figure 3:**
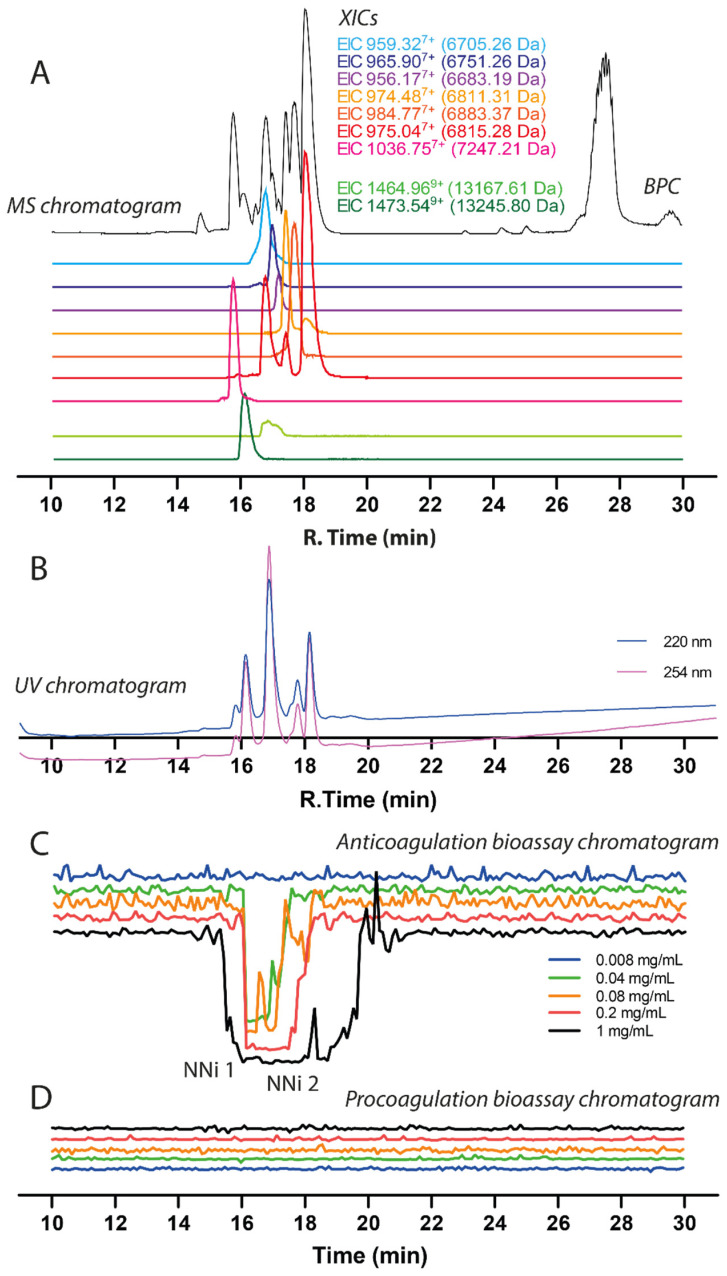
Correlation of LC-UV and MS chromatogram and bioassay chromatogram of *N. nigricollis* venom. (**A**) Base peak chromatogram (BPC), extracted ion currents (XIC), and (**B**) UV chromatogram of *N. nigricollis* venom (1 mg/mL, 50 µL injection volume, post-column split into 1:9 ratio of which the smaller portion went to UV (220 nm and 254 nm recorded); and then to MS detection). (**C**) Superimposed anticoagulation bioassay chromatograms resulting from analyses of serially diluted *N. nigricollis* venom ranging from 1 mg/mL to 0.008 mg/mL (50 µL per injection). (**D**) Superimposed pro-coagulation bioassay chromatograms resulting from analyses of serial diluted *N. nigricollis* venom ranging from 1 mg/mL to 0.008 mg/mL (50 µL per injection). Bioassay chromatograms can be correlated to UV and MS chromatograms. For the pro-coagulation bioassay chromatograms, no pro-coagulation activities were detected.

The chromatographic bioassay profile of 1 mg/mL *N. nigricollis* venom (50 µg injection) was defined by a broad negative peak at the time interval between 15.2–19.8 min (Figure 3, for duplicate experiment see Figure S3). In this anticoagulation time frame, two clear peaks were observed on the parallel UV chromatogram. The negative peak gradually decreased in width upon decreasing the venom quantity injected, and anticoagulant venom activity could still be observed with a loading of just 2 µg venom. At venom concentrations of 0.04 and 0.2 mg/mL venom (2 and 10 µg loaded), the sharp anticoagulant peak shapes allowed for correlation with the peaks observed in the UV trace (and from the MS trace, which was also recorded). Based on this initial concentration series, a concentration of 0.2 mg/mL (10 µg injection) venom was chosen for later assessment of the inhibition of anticoagulant venom toxins via the use of varespladib and marimastat. As an aside, for all of the venom concentrations tested, the procoagulation bioassay chromatograms revealed no procoagulant venom activity, thereby confirming that potent anticoagulant venom activity is not masking any weakly procoagulant venom toxins.

Prior to performing toxin inhibition experiments, we first assigned accurate masses to the venom components exhibiting anticoagulant activity in the plasma coagulation assay. This was done by correlating bioactivity peaks in the bioactivity chromatograms to the corresponding MS chromatogram based on elution time and peak shape of the bioactive peaks. This method proved to be successful for masses up to 15 kDa. However, larger toxins proved to be much more difficult to correlate because masses higher than 20 kDa did not appear in the MS chromatogram, most likely due to a lack of sensitivity of the LC-MS caused by poor ionization or insufficient amounts of the relevant proteins. The resulting mass spectrometry (MS) data is displayed in Figure 3 and consists of the base peak chromatogram (BPC) (shown in black) and nine extracted ion chromatograms (XICs) displayed in light green for acidic PLA_2_, dark green for basic PLA_2_ and red, orange, purple and blue representing 3FTxs. By correlating the negative peak(s) in the bioassay chromatograms with the parallel UV and MS chromatograms, accurate masses of the anticoagulant toxins could be tentatively assigned.

In these analyses, several PLA_2_s closely coeluted and had masses of 13,245.80 Da (coeluting around t_R_ of 16.1 min) and 13,167.61 Da (t_R_ of around 16.8 min). The MS data (i.e., Base peak chromatogram (BPC)) matched in profile with the UV chromatogram data (Figure 3). The peak observed at 27–28 min is the end of the gradient and considered as waste from the column that was detected by MS. The three-finger toxins coeluted around t_R_ 15.7 to 18.5. From the MS data, there were seven 3FTxs detected and five of them were successfully identified. The details of the 3FTxs identified are presented in Table 1. Members of the SVMP toxin family are present at very low concentrations in *N. nigricollis* venom, but due to their relatively high mass these proteins are difficult to protonate and detect with LC-MS. Thus, the LC-MS method in this study was not further used to characterize SVMPs. Bottom-up proteomics methods however do not have this drawback (as the large protease toxins are, after RPLC separation, trypsin digested into smaller peptides prior to MS analysis) and was used to successfully identify several SVMPs.

**Figure 4:**
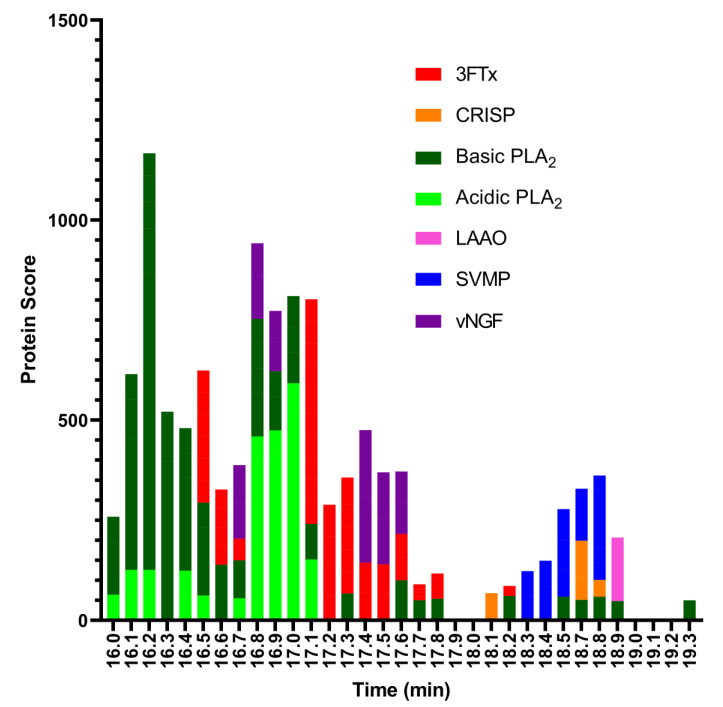
Proteomic annotation of *N. nigricollis* venom using Mascot software and the Swiss-Prot database. The protein score represents the probability that designated proteins, defined by elution time, are present in the sample.

**Figure 5:**
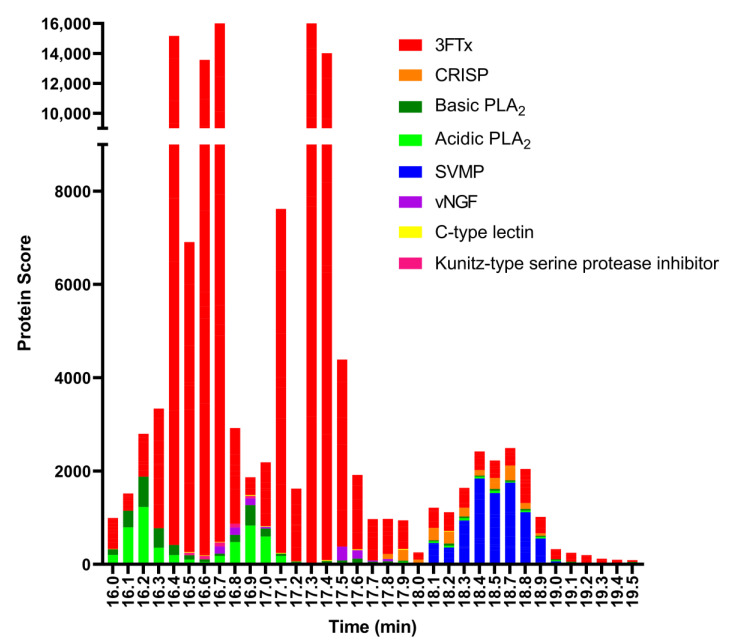
Proteomic annotation of *N. nigricollis* venom using Mascot software and a species-specific venom gland transcriptome-derived database. The protein score represents the probability that designated proteins, defined by elution time, are present in the sample.

For the last step, to identify the coagulopathic venom toxins observed in the plasma coagulation assay after nanofractionation, selected wells containing the anticoagulation bioactive toxins were subjected to tryptic digestion. The trypsin-digested contents of each anticoagulant bioactive well (between retention times 16.0–19.3 min) were analysed using nanoLC-MS/MS and the resulting data were matched against proteins contained in the Swiss-Prot database and in a species-specific database derived from amino acid translations of venom gland transcriptomic data [29]. The protein search from the Swiss-Prot database resulted in 3FTxs, acidic and basic PLA_2_s, L-amino-acid oxidase (LAAO), SVMPs, and venom nerve growth factor (vNGF) (Figure 4). The species-specific database searches resulted in 3FTxs, acidic and basic PLA_2_s, SVMPs, C-type lectin, cysteine-rich secretory protein (CRISP), Kunitz-type serine protease inhibitor, and vNGF (Figure 5). In total, from all wells, we obtained 43 protein matches from the Swiss-Prot database (Figure S1) and 32 from the species-specific transcriptomic database (Figure S2). While the results obtained were complimentary, the discrepancy in number between the two searches is likely the result of the bioactive compounds not having an exact match in the Swiss-Prot database, but instead exhibiting high sequence similarity to several venom toxins deposited in Swiss-Prot from related snake species.

The toxin that eluted around t_R_ 16.1 min matched the basic PLA_2_ PA2B4_NAJNG from *N. nigricollis*, and the toxin that eluted around t_R_ 16.8 min matched the acidic PLA_2_ CM-I (PA2A1_NAJMO) from *N. mossambica*, as determined by searches of the Swiss-Prot database. Searches of the proteomics data against the species-specific database yielded similar results, with the toxins eluted around t_R_ 16.1 min and 16.8 both matching PLA_2_ toxins (transcriptome database ID T0927_T1551_T1904_T2072 and T1049_T1649, respectively). A detailed overview of the PLA_2_s identified from the MS and proteomics data can be found in Table 1.

Many toxins from similar species may also appear as a result, especially for PLA_2_, when using this database for searches. This for example resulted in proteins from other species appearing in the search results, such as PA2A1_NAJMO from *N. mossambica*. The species-specific database is a series of protein sequences that is derived from a venom gland transcriptome study. This study was conducted by the Liverpool School of Tropical Medicine, UK [44,45,46]. The protein sequences in the species-specific database for *N. nigricollis* contained 38 proteins while the protein sequences in the Swiss-Prot database for *N. nigricollis* contained only 8 proteins. The PLA_2_s are under-represented in the species-specific database results (see Figure 5) because the species-specific database predominantly retrieved 3FTxs, which is a consequence of the species-specific database containing predominantly 3FTx protein sequences.

### 2.3. Assessing Inhibition of Nanofractionated Toxins by Varespladib and Marimastat

**Figure 6:**
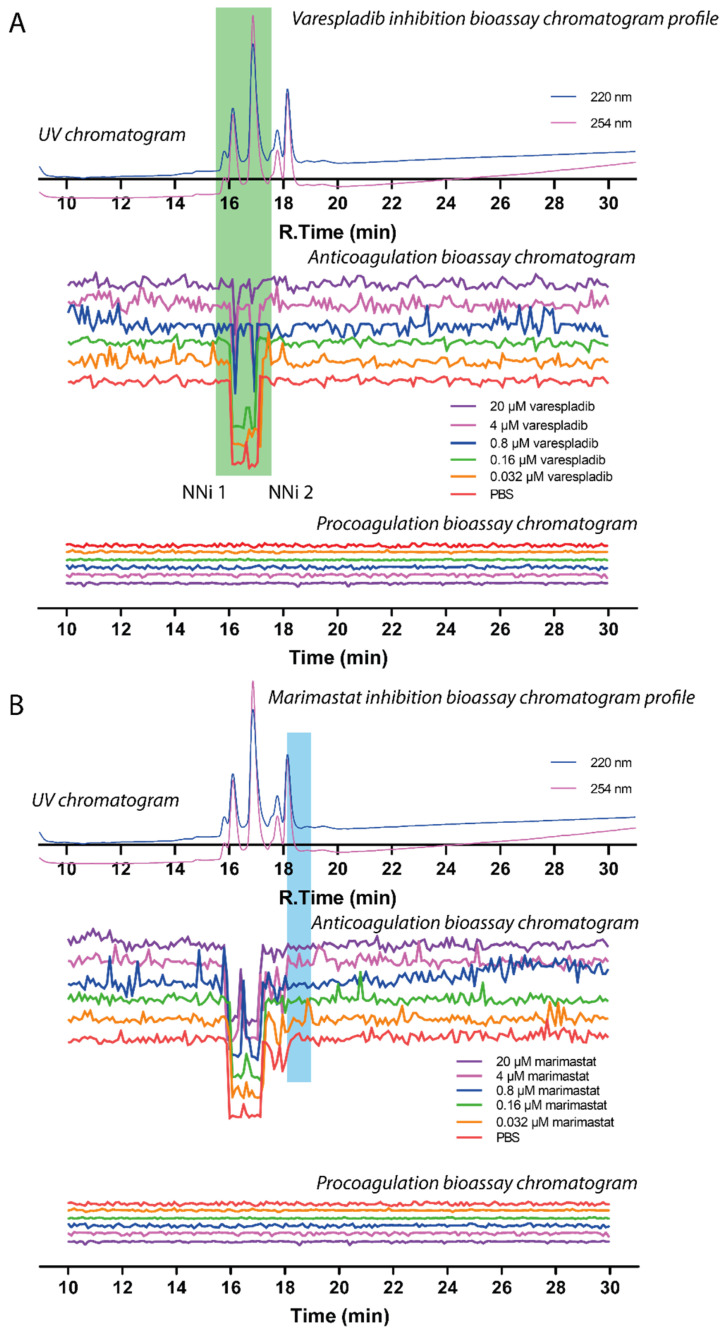
Correlation of UV chromatogram and small molecule inhibition bioassay chromatogram of *N. nigricollis* venom. Superimposed bioassay chromatograms resulting from analyses of *N. nigricollis* venom (0.2 mg/mL, 50 µL injection volume) in the presence of different concentrations of (**A**) varespladib or (**B**) marimastat. Bioassay chromatograms are correlated to UV chromatograms in the figure. For the pro-coagulation bioassay chromatograms, no pro-coagulation activities were detected. Varespladib effectively inhibited anticoagulant venom effects (highlighted in the green rectangle) in a dose dependent manner with inhibitor concentrations ranging from 20 µM to 0.032 µM. Marimastat had no effect on inhibition of anticoagulant venom activity (the retention time where SVMPs are detected using tryptic digestion and nano-LC separation are highlighted in the blue rectangle) at the tested inhibitor concentrations of 20 µM to 0.032 µM.

The two main PLA_2_-mediated anticoagulation peaks observed (PA2B4_NAJNG and PA2A1_NAJMO), within a retention time frame of 15.2–17.2 min, were unsurprisingly not inhibited by the SVMP inhibitor marimastat, but both were dose-dependently inhibited by the PLA_2_ inhibitor varespladib (Figure 6, duplicate experiment see Figures S4 and S5). At the maximum concentration tested (20 µM), both bioactivity peaks were almost fully neutralised by varespladib, as evidenced by notable decreases in both negative peak height and peak width. However, the data presented here clearly show that SVMPs are not associated with the coagulant activity of *N. nigricollis* venom under these conditions, as treatment with marimastat had no effect on the resulting coagulation profiles. Meanwhile, SVMPs were not detected using LC-MS to obtain intact masses (due to high mass/poor ionisation), this toxin family was detected after trypsin digestion of the fractions from the nano-LC separation. This data showed that *N. nigricollis* SVMPs eluted between 18.1 and 18.9 min with no corresponding pro- or anticoagulation activity observed in the bioassay.

### 2.4. Anticoagulant Effects of Isolated and Purified N. nigricollis Toxins

**Figure 7:**
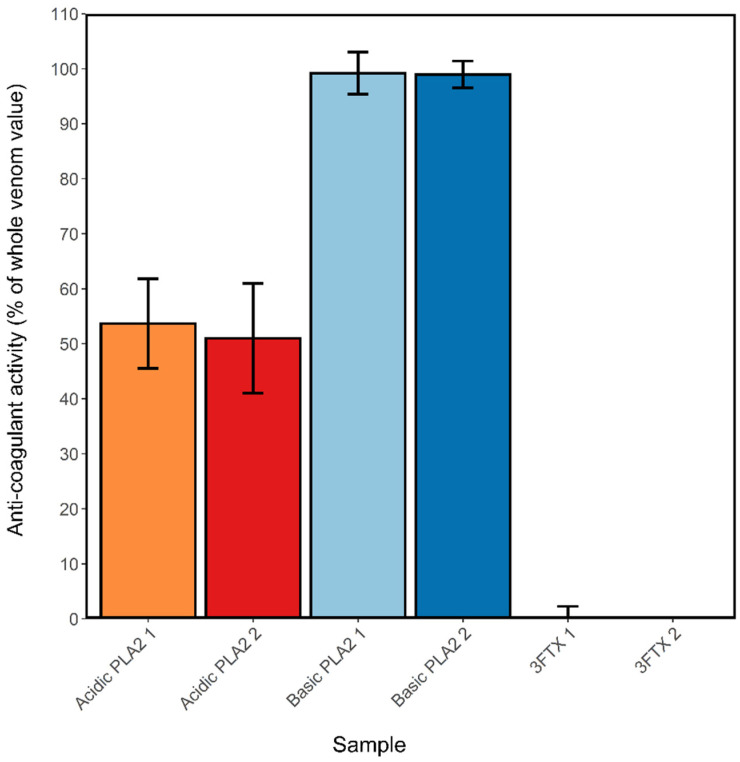
Isolated basic phospholipase A_2_s (PLA_2_s) show anticoagulant activity comparable to that of crude *N. nigricollis* venom. A1-acidic PLA_2_ 1 (mass 13,184 Da), A2-acidic PLA_2_ 2 (mass 13,256 Da), B1-basic PLA_2_ 1 (mass 13,217 Da), B2-basic PLA_2_ 2 (mass 13,248 Da), 3FTx1-three-finger toxin (3FTx) group 1 (mass 6817 Da), 3FTx2–3FTx1–3FTx group 2 (masses 6885, 6818 and 6753 Da). Activity was calculated as the average control area under the curve (AUC) minus the sample AUC, standardized by the mean crude venom AUC, of 2 assays where samples were run at 0.01 mg/mL. Error bars represent the standard error of the mean (SEM).

The ability of varespladib to reduce the anticoagulant activity of *N. nigricollis* venom indirectly suggests that PLA_2_ toxins play a major role in perturbing coagulation, as previously proposed [34]. To investigate this further, the main constituents of *N. nigricollis* venom were purified in mg quantities using gel filtration, cation exchange and reverse-phase chromatography. These formed four groups: Acidic PLA_2_ (two forms with masses 13,184 and 13,256 Da, designated A1 and A2), basic PLA_2_ (two forms with masses 13,217 and 13,247 Da, designated B1 and B2 and the latter equivalent to the XICs displayed in dark green in Figure 3), 3FTx group 1 (mass 6816 Da, equivalent to the XICs displayed in red in Figure 3), and 3FTx group 2 (containing three proteins of mass 6884, 6818 and 6752 Da, likely to be equivalent to the XICs displayed in orange, red and blue respectively in Figure 3). These proteins were tested at 1 µg/mL alongside crude venom across two coagulation assays and the results standardized against the activity of the whole venom sample. Both acidic and basic PLA_2_s exhibited anticoagulant activity (Figure 7), with the acidic types exhibiting approximately 50% of the anticoagulant activity of the whole venom, and the basic types approximately 99% activity. Neither 3FTx groups showed any anticoagulant activity in relation to the crude venom at the concentration used here. This work strongly implicates basic PLA_2_s as being the main toxins responsible for venom-induced anticoagulation in *N. nigricollis* venom, aided by a weaker contributory activity from acidic PLA_2_s.

## 3. Discussion

Our findings demonstrate that *Naja nigricollis* venom displays considerable anticoagulant activity, as shown by coagulation assays utilising bovine plasma, and that despite 3FTxs being the dominant constituents of this venom, this anticoagulant activity observed is caused by PLA_2_ toxins, as exhibited through testing of *N. nigricollis* toxins isolated by nanofractionation or standard chromatographic means. These findings were supported by the reduction in activity observed following application of the small molecule PLA_2_ inhibitor varespladib to crude venom and nanofractionated toxins. These results correlate with previous literature that demonstrate the anticoagulant ability of cobra venoms [34,47] and cobra venom PLA_2_s [23,40,47,48]. Indeed, venom PLA_2_s from the *Naja* genus have previously been found to disrupt haemostatic regulations via the inhibition of both thrombin [25] and platelet aggregation [23,24]. Identification of PLA_2_s as the main anticoagulant components in *N. nigricollis* venom is also consistent with previous nanofractionation and assaying of viper venoms, in which PLA_2_s are the primary anticoagulant toxins in *Bothrops asper*, *Calloselasma rhodostoma*, *Daboia russelii* and *Echis ocellatus* venoms [46].

PLA_2_s in *N. nigricollis* have previously been shown to inhibit the prothrombinase and tenase complexes to prevent coagulation [38,39,40,41], making it unsurprising that they should be identified here as the main anticoagulant components of the venom. Acidic and basic PLA_2_s can both act enzymatically in this respect, acting on the TF-VIIa complex, but in addition basic PLA_2_ can act non-enzymatically through binding to Factor Xa and preventing its binding with Factor Va [38,41,48]. This enhanced anti-coagulation activity of the basic form, compared with that of the acidic, is reflected in the results we obtained from testing purified the PLA_2_s purified from *N. nigricollis* venom (Figure 7).

Despite PLA_2_s being identified as the main venom component responsible for venom-induced coagulopathy caused by *N. nigricollis* venom, the role of 3FTxs and/or the synergistic effects of PLA_2_s and 3FTxs in elapid venoms on haemostasis remains poorly defined. Our results suggest that, at least in the case of *N. nigricollis*, 3FTxs in general appear to have little to no impact on the clotting of plasma. The two 3FTx groups tested here constitute >90% of the 3FTx components of this venom. *Naja* 3FTxs may still play a role in disrupting haemostasis, however, as they have previously been demonstrated to lyse erythrocytes [25,38], and enhance the potency of haemolytic venom activity when in combination with PLA_2_s from *N. mossambica* and *H. haemachatus* venoms [27]. We also found no evidence that the SVMPs in *N. nigricollis* venom perturb coagulation. This is perhaps surprising since SVMPs from *Naja atra* venom have previously been shown to inhibit blood coagulation [49], and that various other P-III SVMPs are known to disrupt coagulation through the activation of prothrombin and/or factor X, cleavage of fibrinogen and/or inhibition of platelet aggregation [50]. It may well be that such activity has not been detected here because of the very low levels of SVMP in the venom of *Naja nigricollis* (<5% of total venom proteins) [29,35] or the likelihood that SVMP activity has been disrupted by the conditions of reverse-phase chromatography utilised in this study.

The ability of the small molecule PLA_2_ inhibitor varespladib to inhibit the anticoagulant activity of *N. nigricollis* venom is consistent with findings from the literature demonstrating the neutralization of PLA_2_-mediated coagulopathy of the venoms of several vipers [51,52,53,54], the elapid *Oxyanurus scutellatus* [51], and many Afro-Asian elapids [34] by varespladib. Varespladib binds to the fatty acid substrate site in PLA_2_ [55], which would explain its inhibition of the enzymatic component of the anti-coagulant action of acidic and basic PLA_2_s, but in this way it may also prevent basic PLA_2_ binding to Factor Xa through steric hindrance, thus also inhibiting this particular mechanism of anti-coagulation. The PLA_2_ His47 amino acid residue, which plays an essential role in the interaction of varespladib and PLA_2_s, was found in both proteins identified by Swiss-Prot as the PLA_2_s PA2B4_NAJNG and PA2A1_NAJMO. In addition to the varespladib-PLA_2_ inhibition observed here and elsewhere relating to anticoagulant venom activity, there is also evidence that varespladib may aid in the inhibition of PLA_2_-mediated neurotoxicity, as seen in the elimination of mortality in mice envenomed by the neurotoxic snakes *Micrurus fulvius*, *Oxyuranus scutellatus* and *Vipera berus nikolskii* [56,57,58,59,60]. Combined, these data suggest that varespladib is a highly promising future treatment option for tackling snakebite.

## 4. Materials and Methods

### 4.1. Chemical and Biological Reagents

Water was purified with a milli-Q Plus system (Millipore, Amsterdam, The Netherlands). DMSO was supplied by Redel-de-Haen (Zwijndrect, The Netherlands). Acetonitrile (ACN) was obtained from Concord, NC, USA; UPLC/MS grade). Formic acid (FA, MS grade) and acetic acid (AA) were purchased from Biosolve (Valkenswaard, The Netherlands). CaCl_2_, K_2_HPO_4_, KH_2_PO_4_, NH_4_HCO_3_, Phosphate Buffer Saline (PBS) tablets, and other salts used for buffer preparation were of analytical grade and purchased from trusted suppliers (Merck, Kenilworth, UK; Fluka, Bucharest, Romania; or Sigma-Aldrich, Darmstadt, Germany). The PLA_2_ inhibitor varespladib, SVMP inhibitor marimastat, and iodoacetamide were purchased from Sigma-Aldrich.

Lyophilized *N. nigricollis* venom pooled from multiple animals of Nigerian origin was provided by the Centre for Snakebite Research and Interventions Herpetarium (Liverpool School of Tropical Medicine, Liverpool, UK) and stored long-term at 4 °C. Stock solutions of the crude venom (5.0 ± 0.1 mg/mL) were prepared in water before analysis, and then aliquoted and stored at −20 °C until use. Bovine plasma (500 mL bottles) used in the nanofractionation assays was purchased from Biowest (Nuaille, France), that for assay of venom and purified toxins was provided by Equitech-Bio (SBPUC35-0100). The plasma was defrosted in a water bath at 37 °C, centrifuged at slow speed, and then rapidly aliquoted (approximately 10 mL in 15 mL falcon tubes) and stored at −80 °C prior to use. Lyophilized trypsin Gold^TM^ (mass spectrometry grade) enzyme was purchased from Promega Corporation, Madison, WI, USA. The trypsin was reconstituted in 50 mM acetic acid to obtain a 1 μg/μL concentration, which was aliquoted and stored at −80 °C until use.

### 4.2. Coagulopathic Activity of Crude N. nigricollis Venom and Assessment of Inhibition with Varespladib and Marimastat

Plasma clotting assays on crude *N. nigricollis* venom and toxins purified by chromatography were performed on citrated bovine plasma using previously described protocols [61]. Thawed, plasma was centrifuged at 2000× *g* for 4 min to remove precipitates. For crude venom assays, 10 µL of *N. nigricollis* venom at concentrations of 1 µg/mL, 10 µg/mL and 100 µg/mL in PBS (final assay concentrations of 0.002, 0.02 and 0.2 µg/mL respectively) was pipetted in quadruplicate onto a Greiner Bio-One clear 384-well microplate, while 10 µL PBS was pipetted into control wells. To all wells, a 20 uL solution of 20 mM calcium chloride was added using a Thermo Scientific™ Multidrop™ 384 Labsystems multidrop pipettor (Oxford, UK). The multidrop was then flushed with deionized water before being used to add 20 µL of plasma into each well. Plates were read kinetically at a wavelength of 595 nm and a temperature of 25 °C using a FLUOstar Omega plate reader (Aylesbury, UK), with run time set to 110 cycles at a cycle time of 80 s. Clotting activity was measured as the area under the absorption curve (AUC) from 0 to 100 min, calculated by the Graphpad Prism v. 8.0 software.

For assays involving inhibitors, 5 well types were set up: (i) venom + inhibitor, consisting of 1 µL of crude 100 µg/µL *N. nigricollis* venom (final concentration in the assay 2 µg/mL), 3.75 µL of the SVMP inhibitor marimastat (Sigma-Aldrich, M2699-5MG) or PLA_2_ inhibitor varespladib (Sigma-Aldrich, SML1100-5MG) at 2 mM or 6 mM (final concentration in the assay, 150 and 450 µM), and 5.25 µL PBS, (ii) venom control, containing 1 µL of crude 100 µg/µL *N. nigricollis* venom and 9 µL PBS, (iii) inhibitor control: 3.75 µL of marimastat or varespladib at 2 mM or 6 mM (final concentration in the assay, 150 and 450 µM), and 6.25 µL PBS, (iv) Dimethylsulfoxide (DMSO) control: 3.75 µL of the reconstituting solvent for the inhibitors, DMSO, plus 6.25 µL PBS, (v) negative control: 10 µL PBS. Wells were pipetted into a 384-well plate in quadruplicate after being incubated for 30 min at 37 °C, followed by the addition of 20 µL 20 mM calcium chloride and 20 µL bovine plasma. The absorbance of the plasma was then read at 595 nm on a FLUOstar Omega plate reader at 25 °C.

### 4.3. HPLC-MS-Nanofractionation and Simultaneous Bioassay of N. nigricollis Venom

Separation of venom toxins for the post-column bioassays was carried out by LC with high-resolution nanofractionation in parallel to MS analysis. Samples were injected with a Shimadzu SIL-20A autosampler, and the separation was performed with an LC system controlled via Shimadzu Lab Solution software. The gradient was set using a binary Shimadzu LC-20AB pump (A and B) at a total flow rate of 0.5 mL/min. Mobile phase A was water-ACN-FA (98:2:0.1, *v*/*v*/*v*) and mobile phase B was water-ACN-FA (2:98:0.1, *v*/*v*/*v*). The following gradient was used: 0% to 10% B (10 min), 10% to 95% B (20 min), 95% B (2 min), 90% to 0% B (7 min), 0% B (2 min). A 100 × 4.6 mm ID analytical column packed with Xbridge BEH300 reversed-phase C18 material (3.5 μm) was used for separation. The column eluate was split in a 1:9 ratio using a low-dead-volume flow splitter. The smaller flow part after the split (0.05 mL/min) was directed via a Shimadzu SPD 20A UV-Vis detector (’s-Hertogenbosch, The Netherlands) with dual-wavelength (220 nm and 254 nm) measurement to a Bruker Maxis HD Mass Spectrometer (Bruker Daltonics, Bremen, Germany). The larger part of the eluate split was fractionated (6 s/well) onto transparent 384-well plates (Grenier Bio-One, Alphen aan den Rijn, The Netherlands) by use of a FractioMateTM FRM100 nanofraction collector (SPARK-Holland & VU, Netherlands, Emmen & Amsterdam) controlled by FractioMator software or by a modified Gilson 235P autosampler controlled by in-house written Ariadne software. After nanofractionation, the plates were vacuum centrifuged overnight to dryness at room temperature using a Christ Rotational Vacuum Concentrator RVC 2-33 CD Plus (Salm en Kipp, Breukelen, The Netherlands) with integrated cooling trap operating at −80 °C. The plates were then stored at −80 °C until bioassayed or proteomics experiments were performed. For mass analysis, the Maxis HD mass spectrometer was equipped with an electrospray ionization source (ESI) and operated in positive ion mode. The parameters of the ESI source were: source temperature, 180 °C; desolvation temperature of 180 °C; capillary voltage of 4500 V; a gas flow of 4 L/min. The monitored mass range was *m*/*z* 500–3000 with a data-sampling time of 1 s. Protein masses (Da) were calculated using Data Analysis 5.0 (Bruker, Darmstadt, Germany) software.

The venom dilution test was important for determining optimum venom concentration for subsequent analyses, for which the venom concentration that exhibited sharp bioassay peaks was chosen. These sharp peak(s) were desired for the subsequent small molecule inhibitory assays as in this way, the effect of varespladib and marimastat could most sensitively and effectively be assessed. A series of venom concentrations (1 mg/mL; 0.2 mg/mL; 0.08 mg/mL; 0.04 mg/mL; and 0.008 mg/mL) was made by diluting 5 mg/mL venom stock solution with Milli-Q water. These venom solutions (50 µL) were injected into the HPLC-MS system equipped with a nanofractionation module. Nanofractionated well plates were freeze-dried, after which the coagulation activity assay was performed on each plate. The resulting bioassay chromatogram from the venom dilution experiments were superimposed on UV and MS traces.

The resulting bioassay chromatograms for measuring any evidence of procoagulation and anticoagulation were generated and processed as follows: measurement of each plate consisted of a kinetic loop measurement with 80 readings of the complete plate at an interval of 15 s per reading. For plotting the procoagulant bioassay chromatograms, the average rate of the coagulation curve measured for each well from reading 1 to 15 was used from the kinetic loop data. This was achieved by plotting the procoagulation bioassay chromatograms as bioassay signal (y-axis) against fraction time (x-axis), which gave resembled bioassay chromatograms. This resulted in positive peaks for eluted toxins with pro-coagulatory effects (though none were observed). For the anticoagulation bioassay chromatograms, the last absorbance reading (i.e., reading 80) of each well was plotted as the y-axis in combination with fractionation time on the x-axis. Eluted venom toxins with an anticoagulation effect showed up as negative peaks in these bioassay chromatograms.

### 4.4. Assessment of Inhibition of Nanofractionated Anticoagulant Toxins with Varespladib and Marimastat

All measurements of coagulation activity were carried out in freeze-dried 384-well plates in duplicate. The plates were first incubated at room temperature for assay preparation. To initiate the coagulation process, the mixing of plasma with calcium chloride solution was carried out by transferring 20 μL of 20 mM calcium chloride solution to the freeze-dried plate with 20 µL citrated bovine plasma using the Thermo Scientific Multidrop^TM^ pipetting robot. Serial dilutions (0.16 µM; 0.8 µM; 4 µM; 20 µM; and 100 µM) of varespladib and marimastat were made in PBS. Next, 10 µL of each inhibitor solution was added to rows 8-18 of nanofractionated plates prior to further bioassay preparations and measurements. Additionally, PBS was used as a negative control in row 4–7 and in rows 19–22. The plate was spun down by centrifuging for 2 min and then incubated at room temperature for 30 min with gentle shaking. The CaCl_2_ was added to corresponding well to perform the coagulation assay, resulting in final assay concentrations of varespladib and marimastat of 0.032 µM; 0.16 µM; 0.8 µM; 4 µM; and 20 µM. Thereafter, plate absorbance was measured at a wavelength of 595 nm by a Thermo Scientific Varioskan Lux^TM^ Plate reader using SkanIt 4.1 software. Measurements were performed in one kinetic loop consisting of 80 readings, within 1.5 h. Two forms of data processing were carried out to produce different representations of the coagulation chromatograms: a single reading at 80th reading (1.5 h) for anticoagulation, the slope of a reading range for determining any pro-coagulation [62].

### 4.5. In-Solution Tryptic Digestions from Nanofractionated 384-Well Plates

Vacuum centrifuged nanofractionated well plates were defrosted at room temperature and the contents of the wells whose contents were selected for digestion were dissolved in 40 μL milli-Q water. The plates were then spun down at 1000× *g* for 30 s using a plate centrifuge (Eppendorf^®^ Centrifuge 5810R), followed by shaking at 60 RPM for 30 min on a plate shaker (IKA^®^ KS 4000 IC Control). Digestion buffer was prepared by mixing reducing agent (0.5% (*v*/*v*) β-mercaptoethanol in milli-Q water and AmmBi buffer pH 8.2 (25 mM ammonium bicarbonate) at a 1:9 ratio. For each sample, an Eppendorf tube was filled with 50 μL digestion buffer, before the addition of 30 μL of re-dissolved venom protein sourced from each well of the well plate, followed by vortexing for 10 s. The samples were then incubated at 95 °C for 10 min for reduction using a dry block heating thermostat (Biosan^®^ Bio TDB-100). Thereafter, 9 μL of alkylation agent (100 mM iodoacetamide) was added to each sample followed by incubation in the dark at room temperature for 30 min. Trypsin stock solution was diluted with AmmBi buffer pH 8.2 to obtain a 0.1 μg/μL working concentration and 5 μL added to each Eppendorf tube, followed by vortexing and incubation at 37 °C for 3 h. An additional 5 μL of trypsin solution was then added to each sample prior to incubation at 37 °C overnight. Subsequently, 10 μL of 5% formic acid solution was added to each tube to terminate the digestion process. The resulting samples were transferred to autosampler vials and analysed by nanoLC-ESI-MS/MS according to method in Section 4.6.

### 4.6. Proteomics of Coagulopathic Venom Toxins

For proteomics analysis, an Ultimate 3000 nano HPLC module (Thermo Scientific, Waltham, MA, USA) coupled to a Bruker Tims-TOF Mass Spectrometer (Bruker Daltonics, Bremen, Germany) was used. Samples were injected with a WPS-3000(RS) autosampler, and nanoLC separations were performed with a nanoLC system controlled via Chromeleon 7.2 SR4 MUb software. The gradient used was set using a nanoLC binary pump (A and B) at a total flow rate of 0.5 µL/min. Mobile phase A was water-FA (100:0.1 *v*/*v*), and mobile phase B was water-ACN-FA (20:80:0.1, *v*/*v*/*v*). The system was also equipped with a loading pump, for which solvent water-ACN-FA (99:1:0.05 *v*/*v*/*v*) was used. The following gradient was used: 1% B (10 min), 1%–20% B (5 min), 20%–50% B (30 min), 50%–85% B (1 min), 85% B (5 min) 85%–1% B (0.5 min), 1% B (9.5 min). For sample trapping, an Acclaim PepMap 100 reversed-phase C18 trapping column (particle diameter 5 µm and column dimensions of 5 × 0.3 mm) was used. An Acclaim PepMap 100 reversed-phase C18 analytical column (particle diameter 2 µm and column dimensions of 150 × 0.75 mm), was used to subsequently separate the peptides in the samples. Both the analytical column and the trapping column were placed in a column oven of which the temperature was set at 45 °C. The column eluate was transferred to a Bruker TIMS-TOF Mass Spectrometer, equipped with a captive spray ionization (CSI) source in positive ion mode. The parameters of the CSI source were: source temperature, 150 °C; desolvation temperature, 180 °C; capillary voltage, 1300 V; gas flow, 3 L/min. The monitored mass range was m/z 300–3000 with a data-sampling time of 0.5 s. The collision energy was 10 eV with pre-pulse storage 10 µs.

The raw MS/MS data from the nanoLC-ESI-MS/MS analyses were extracted using Bruker Data Analysis 5.0 and converted into deconvoluted extracted ion chromatograms (XICs). These deconvoluted XICs were then converted into Mascot generic format (MGF) files using Data Analysis 5.0 and an in-house written script for automatization of this process. The script contained instruction and parameters for deconvoluting the spectrum included. The resulting MGF files were uploaded to Mascot to be used in database searches against two different databases: the Swiss-Prot database and a species-specific database for *N. nigricollis* from LSTM, UK [63]. The parameter used at Mascot searches depends on the protein digestion method. To achieve consistent results, this following parameter was used: (i) since iodoacetamide was used as an alkylating agent then fixed modification: carbamidomethyl (C) was chosen (add 34 Da to methionine residue); (ii) the variable modification: amidated (C-term) and methionine oxidation (M), (iii) peptide tolerance of ±0.1% and MS/MS tolerance of ±0.05 Da, (iv) peptide charge of +1, +2, and +3.

All wells of proteins nanofractionated between retention times 16.0–19.3 min were analysed (nanofractionation resolution 6 s) using proteomics approaches. The two database searches using the same proteomics data yielded complementary results. The species-specific database has the advantage that it exactly matches the searched proteomics data with the corresponding transcriptomics data. This way only exactly matching results are retrieved. The species-specific database contains 21 three-finger toxin entries, but it does not provide additional valuable information that is available in Swiss-Prot. The Swiss-Prot data base can provide extra information such as location of disulphide bridge, sequence of signal-peptide, and protein mass. Therefore, in this study proteomics searches were done using both databases in order to achieve results from the two database searches that are complementary to each other. The species-specific database was also able to detect minor venom toxins that were not picked up by the LC-MS measurements (such as CRISP, NGF, SVMPs). On the other hand, the Swiss-Prot database provided cross-species searches which were useful for gaining a better understanding of the active toxin enzymes eluting at the anticoagulation bioactivity peak retention times.

### 4.7. Chromatographic Isolation of N. nigricollis Venom Components

To confirm the proteomic identification of venom components responsible for anticoagulant activity in *N. nigricollis* venom, the major venom constituents (3FTxs and PLA_2_s) were isolated using standard chromatographic methods. For the first step in the purification, 40 mg of *N. nigricollis* [Nigeria] venom dissolved in PBS was subjected to gel filtration chromatography on 120 mL column of Superdex 75 [Cytiva]. The column was operated at 1 mL/min using PBS as buffer. This separated the high molecular weight material (SVMPIII and LAAO) from the 3FTx and PLA_2_s; the latter two toxin groups eluted together as a large single peak. This combined 3FTx, PLA_2_ material was dialysed against 50 mM sodium phosphate, pH 5.8 and loaded on to a 4.7 mL cation exchange column (Hi-Screen SP, Cytiva) and the toxins were then separated with a 0–0.7 M gradient of NaCl in 50 mM sodium phosphate, pH 5.8. The main toxins eluted in the order acidic PLA_2_ (two forms) 3FTx group 1, 3FTx group 2 (containing three forms) and finally basic PLA_2_ (two forms). Following dialysis into PBS, the 3FTx fractions were used directly for anti-coagulation studies. Acidic and basic PLA_2_s were further purified using reverse-phase chromatography. Thus, 1 mg of each was separately loaded onto a Phenomenex Jupiter C4 column [250 × 4.6 mm] and then eluted using a 0–70% gradient of acetonitrile in 0.1% TFA. The solvent was removed by rotary evaporation, avoiding complete drying, and then reconstituted in PBS. Purified proteins were run on two plasma assays as per 4.2. at 1 μg/mL against 2 μg/mL (final concentrations) crude *N. nigricollis* venom in replicates of 8. Anticoagulant activity of isolated toxins was determined as a percentage of that of the crude venom.

## Figures and Tables

**Table 1:** Overview of tentatively assigned venom toxins associated with the observed anticoagulation peaks. The table shows *m/z* values, including charge state measured for each *m/z* value of intact toxins observed in MS, their retention times, and the calculated accurate mass for each toxin. Moreover, proteomics derived Mascot (Swiss-Prot) protein identification and Mascot derived corresponding masses are shown, as are matches to the species-specific venom gland transcriptome databases. Hits were obtained by database searches of nanoLC-MS/MS data measured from tryptic digests of the respective venom toxins after nanofractionation and collecting these venom toxins from their respective wells in which they were fractionated.

Retention Time (min)	*m*/*z* Value (Charge)	MS Accurate Mass (Da)	Mascot Protein Hits (Protein Identification)	Mascot Exact Mass (Da)	Species-Specific Database Hits	Toxin Class
15.7	1036.75(+7)	7247.21	Three-finger toxin	N.A.	N.A.	3FTx
		13,217	Basic Phospholipase A_2_ (B1) **	N.A.	N.A.	PLA_2_
16.1	1473.54 (+9)	13,245.80	PA2B4_NAJNG (*Naja nigricollis*) Phospholipase A_2_ Basic (B2) **	13,244.90	T0927_T1551_T1904_T2072	PLA_2_
		13,184	Acidic Phospholipase A_2_ (A1) **	N.A.	N.A.	PLA_2_
		13,256	Acidic Phospholiase A_2_ (A2) **	N.A.	N.A.	PLA_2_
16.8	1464.96 (+9)	13,167.61	PA2A1_NAJMO (*Naja mossambica*) Acidic Phospholipase A_2_ CM-I	13,195.75	T1049_T1649	PLA_2_
16.8	959.32(+7)	6705.26	3SA4_NAJMO (*Naja mossambica*) Cytotoxin 4	6702.34	T1903_R_6_8306_350	3FTx
17.0	965.90(+7)	6751.26	3SA9_NAJNA (*Naja naja*) Cytotoxin 9 (3FTx group 2) **	6750.36 *	T0802_R_6_4763_L_609	3FTx
17.2	956.17(+7)	6683.19	3SAN_NAJNG (*Naja nigricollis*) Naniproin	6682.41	T2051_R_3_6355_L_312	3FTx
17.4	974.48(+7)	6811.31	3SA5_NAJAT (*Naja atra*) Cytotoxin 5	6810.35 *	T2073_R_2_4857_L_300	3FTx
17.7	984.77(+7)	6883.37	Three-finger toxin (3FTx group 2) **	N.A.	N.A.	3FTx
18.0	975.04(+7)	6815.28	3SA1_NAJPA (*Naja pallida*) Cytotoxin 1 (3FTx group 2) **	6814.31	T0939_T1746_R_11_8132_L_555	3FTx
		6817	Three-finger toxin (3FTx group 1) **	N.A.	N.A	3FTx

* calculated without signal peptide, ** protein was obtained from purified sample, N.A. = not applicable.

## Data Availability

Data is available upon request.

## Supplementary Materials

The following are available online at https://www.mdpi.com/article/10.3390/toxins13050302/s1, Figure S1: Detailed results of proteomics searches using Mascot software against the Swiss-Prot Database to identify anticoagulant venom proteins, Figure S2: Detailed results of proteomics searches using Mascot software against the *N. nigricollis* species-specific venom gland transcriptome-derived database. Figure S3: Duplication results of superimposed pro- and anticoagulation bioassay chromatograms resulting from analyses of serial diluted *N. nigricollis* venom ranging from 1 mg/mL to 0.008 mg/mL (50 µL per injection). Figure S4: Duplication results of superimposed bioassay chromatograms resulting from analyses of *N. nigricollis* venom (0.2 mg/mL, 50 µL injection volume) in the presence of different concentrations of varespladib. Figure S5: Duplication results of superimposed bioassay chromatograms resulting from analyses of *N. nigricollis* venom (0.2 mg/mL, 50 µL injection volume) in the presence of different concentrations of marimastat.

## References

[1] Kasturiratne A., Wickremasinghe A.R., De Silva N., Gunawardena N.K., Pathmeswaran A., Premaratna R., Savioli L., Lalloo D.G., De Silva H.J. (2008). The global burden of snakebite: A literature analysis and modelling based on regional estimates of envenoming and deaths. PLoS Med..

[2] Gutiérrez J.M., Calvete J.J., Habib A.G., Harrison R.A., Williams D.J., Warrell D.A. (2017). Snakebite envenoming. Nat. Rev. Dis. Prim..

[3] Chippaux J.P. (1998). Snake-bites: Appraisal of the global situation. Bull. World Health Organ..

[4] Fox S., Rathuwithana A.C., Kasturiratne A., Lalloo D.G., de Silva H.J. (2006). Underestimation of snakebite mortality by hospital statistics in the Monaragala District of Sri Lanka. Trans. R. Soc. Trop. Med. Hyg..

[5] Longbottom J., Shearer F.M., Devine M., Alcoba G., Chappuis F., Weiss D.J., Ray S.E., Ray N., Warrell D.A., Bill F. (2018). Vulnerability to snakebite envenoming: A global mapping of hotspots. Lancet.

[6] Warrell D.A., Meier J., White J. (2008). Clinical Toxicology of Snakebite in Asia. Handbook of Clinical Toxicology of Animal Venoms and Poisons.

[7] Warrell D.A., Meier J., White J. (2008). Clinical Toxinology of Snakebite in Africa and the Middle East/Arabian Penninsula. Handbook of Clinical Toxicology of Animal Venoms and Poisons.

[8] Blaylock R.S., Lichtman A.R., Potgieter P.D. (1985). Clinical manifestations of Cape cobra (*Naja nivea*) bites. A report of 2 cases. S. Afr. Med. J..

[9] Warrell D.A., Barnes H.J., Piburn M.F. (1976). Neurotoxic effects of bites by the Egyptian cobra (*Naja haje*) in Nigeria. Trans. R. Soc. Trop. Med. Hyg..

[10] Zouari N., Choyakh F. (1995). Les effets neurotoxiques du venin de cobra (*Naja haje haje*) sur la jonction neuromusculaire. Étude électroclinique de deux cas en Tunisie. Neurophysiol. Clin..

[11] Strover H.M. (1973). Observations on two cases of snake-bite by Naja nigricollis ss mossambica. Cent. Afr. J. Med..

[12] Tilbury C.R. (1982). Observations on the bite of the Mozambique spitting cobra (*Naja mossambica mossambica*). S. Afr. Med. J..

[13] Warrell D.A., Greenwood B.M., Davidson N.M., Ormerod L.D., Prentice C.R. (1973). Necrosis, haemorrhage and complement depletion following bites by the spitting cobra (*Naja nigricollis*). Q. J. Med..

[14] Wüster W., Crookes S., Ineich I., Mane Y., Pook C.E., Trape J.F., Broadley D.G. (2007). The phylogeny of cobras inferred from mitochondrial DNA sequences: Evolution of venom spitting and the phylogeography of the African spitting cobras (Serpentes: Elapidae: Naja nigricollis complex). Mol. Phylogenet. Evol..

[15] Pugh R.N.H., Theakston D.G. (1980). Incidence and Mortality of Snake Bite in Savanna Nigeria. Lancet.

[16] Pugh R.N.H., Theakston R.D.G., Reid H.A., Bhar I.S. (1980). Malumfashi Endemic Diseases Research Project, XIII. Ann. Trop. Med. Parasitol..

[17] Habib A.G., Gebi U.I., Onyemelukwe G.C. (2001). Snake bite in Nigeria. Afr. J. Med. Med. Sci..

[18] Rivel M., Solano D., Herrera M., Vargas M., Villalta M., Segura Á., Arias A.S., León G., Gutiérrez J.M. (2016). Pathogenesis of dermonecrosis induced by venom of the spitting cobra, *Naja nigricollis*: An experimental study in mice. Toxicon.

[19] Méndez I., Gutiérrez J.M., Angulo Y., Calvete J.J., Lomonte B. (2011). Comparative study of the cytolytic activity of snake venoms from African spitting cobras (*Naja spp*., Elapidae) and its neutralization by a polyspecific antivenom. Toxicon.

[20] Dutta S., Sinha A., Dasgupta S., Mukherjee A.K. (2019). Binding of a *Naja naja* venom acidic phospholipase A 2 cognate complex to membrane-bound vimentin of rat L6 cells: Implications in cobra venom-induced cytotoxicity. Biochim. Biophys. Acta Biomembr..

[21] Slagboom J., Kool J., Harrison R.A., Casewell N.R. (2017). Haemotoxic snake venoms: Their functional activity, impact on snakebite victims and pharmaceutical promise. Br. J. Haematol..

[22] McCleary R.J.R., Kini R.M. (2013). Snake bites and hemostasis/thrombosis. Thromb. Res..

[23] Sundell I.B., Rånby M., Zuzel M., Robinson K.A., Theakston R.D.G. (2003). In vitro procoagulant and anticoagulant properties of *Naja naja naja* venom. Toxicon.

[24] Dutta S., Gogoi D., Mukherjee A.K. (2015). Anticoagulant mechanism and platelet deaggregation property of a non-cytotoxic, acidic phospholipase A2 purified from Indian cobra (*Naja naja*) venom: Inhibition of anticoagulant activity by low molecular weight heparin. Biochimie.

[25] Osipov A.V., Filkin S.Y., Makarova Y.V., Tsetlin V.I., Utkin Y.N. (2010). A new type of thrombin inhibitor, noncytotoxic phospholipase A2, from the *Naja haje* cobra venom. Toxicon.

[26] Jiang M.-S., Fletcher J.E., Smith L.A. (1989). Factors influencing the hemolysis of human erythrocytes by cardiotoxins from *Naja naja kaouthia* and *Naja naja atra* venoms and a phospholipase A2with cardiotoxin-like activities from *Bungarus fasciatus* venom. Toxicon.

[27] Banerjee Y., Mizuguchi J., Iwanaga S., Kini R.M. (2005). Hemextin AB complex, a unique anticoagulant protein complex from *Hemachatus haemachatus* (African Ringhals cobra) venom that inhibits clot initiation and factor VIIa activity. J. Biol. Chem..

[28] Louw A.I., Visser L. (1978). The synergism of cardiotoxin and phospholipase A2 in hemolysis. BBA Biomembr..

[29] Kazandjian T.D., Petras D., Robinson S.D., van Thiel J., Greene H.W., Arbuckle K., Barlow A., Carter D.A., Wouters R.M., Whiteley G. (2021). Convergent evolution of pain-inducing defensive venom components in spitting cobras. Science.

[30] Tasoulis T., Isbister G.K. (2017). A review and database of snake venom proteomes. Toxins.

[31] Kini R.M. (2006). Serine proteases affecting blood coagulation and fibrinolysis from snake venoms. Pathophysiol. Haemost. Thromb..

[32] Phillips D.J., Swenson S.D., Markland F.S.J., Mackessy S.P. (2010). Thrombin-Like Snake Venom Serine Proteinases. Handbook of Venoms and Toxins of Reptiles.

[33] Jagadeesha D.K., Shashidharamurthy R., Girish K.S., Kemparaju K. (2002). A non-toxic anticoagulant metalloprotease: Purification and characterization from Indian cobra (*Naja naja naja*) venom. Toxicon.

[34] Bittenbinder M.A., Zdenek C.N., Op Den Brouw B., Youngman N.J., Dobson J.S., Naude A., Vonk F.J., Fry B.G. (2018). Coagulotoxic cobras: Clinical implications of strong anticoagulant actions of african spitting naja venoms that are not neutralised by antivenom but are by LY315920 (varespladib). Toxins.

[35] Petras D., Sanz L., Segura Á., Herrera M., Villalta M., Solano D., Vargas M., León G., Warrell D.A., Theakston R.D.G. (2011). Snake venomics of African spitting cobras: Toxin composition and assessment of congeneric cross-reactivity of the Pan-African EchiTAb-Plus-ICP antivenom by antivenomics and neutralization approaches. J. Proteome Res..

[36] Evans H.J. (1981). Cleavage of the Aa-chain of fibrinogen and the A-polymer of fibrin by the venom of the spitting cobra (*Naja nigricollis*). Biochem. Biophys. Acta.

[37] Mackay N., Ferguson J.C., Mcnicol G.P. (1969). Effects of three cobra venoms on blood coagulation, platelet aggregation, and fibrinolysis. J. Clin. Pathol..

[38] Stefansson S., Kini R.M., Evans H.J. (1990). The Basic Phospholipase A2 from *Naja nigricollis* Venom Inhibits the Prothrombinase Complex by a Novel Nonenzymatic Mechanism. Biochemistry.

[39] Kini R.M., Evans H.J. (1995). The role of enzymatic activity in inhibition of the extrinsic tenase complex by phospholipase A2 isoenzymes from *Naja nigricollis* venom. Toxicon.

[40] Stefansson S., Kini R.M., Evans H.J. (1989). The inhibition of clotting complexes from the extrinsic coagulation cascade by the phospholipase A2 isoenzymes from *Naja nigricollis* venom. Thromb. Res..

[41] Kerns R.T., Kini R.M., Stefansson S., Evans H.J. (1999). Targeting of venom phospholipases: The strongly anticoagulant phospholipase A2 from Naja nigricollis venom binds to coagulation factor Xa to inhibit the prothrombinase complex. Arch. Biochem. Biophys..

[42] Kini R.M., Evans H.J. (1988). Mechanism of platelet effects of cardiotoxins from *Naja nigricollis crawshawii* (spitting cobra) snake venom. Thromb. Res..

[43] Still K.B.M., Slagboom J., Kidwai S., Xie C., Zhao Y., Eisses B., Jiang Y., Vonk F.J., Somsen G.W., Casewell N.R. (2020). Development of high-throughput screening assays for profiling snake venom Phospholipase A2 activity after high-resolution chromatographic fractionation. Toxicon.

[44] Ainsworth S., Petras D., Engmark M., Süssmuth R.D., Whiteley G., Albulescu L.O., Kazandjian T.D., Wagstaff S.C., Rowley P., Wüster W. (2018). The medical threat of mamba envenoming in sub-Saharan Africa revealed by genus-wide analysis of venom composition, toxicity and antivenomics profiling of available antivenoms. J. Proteom..

[45] Davidson T.M., Eisner J. (1996). Colective Review: United States coral snakes. Wilderness Environ. Med..

[46] Slagboom J., Mladić M., Xie C., Kazandjian T.D., Vonk F., Somsen G.W., Casewell N.R., Kool J. (2020). High throughput screening and identification of coagulopathic snake venom proteins and peptides using nanofractionation and proteomics approaches. PLoS Negl. Trop. Dis..

[47] Bittenbinder M.A., Dobson J.S., Zdenek C.N., op den Brouw B., Naude A., Vonk F.J., Fry B.G. (2019). Differential destructive (non-clotting) fibrinogenolytic activity in Afro-Asian elapid snake venoms and the links to defensive hooding behavior. Toxicol. Vitr..

[48] Kini R.M. (2005). Structure-function relationships and mechanism of anticoagulant phospholipase A2 enzymes from snake venoms. Toxicon.

[49] Sun Q., Wang C., Li Y., Bao J. (2020). Toxicon Inhibition of platelet aggregation and blood coagulation by a P-III class metalloproteinase purified from *Naja atra* venom. Toxicon.

[50] Olaoba O.T., dos Santos P.K., Selistre-de-Araujo H.S., Ferreira de Souza D.H. (2020). Snake Venom Metalloproteinases (SVMPs): A structure-function update. Toxicon X.

[51] Xie C., Albulescu L.-O., Still K., Slagboom J., Zhao Y., Jiang Z., Somsen G., Vonk F., Casewell N., Kool J. (2020). Varespladib inhibits the phospholipase A2 and coagulopathic activities of venom components from haemotoxic snakes. Biomedicines.

[52] Youngman N.J., Walker A., Naude A., Coster K., Sundman E., Fry B.G. (2020). Varespladib (LY315920) neutralises phospholipase A2 mediated prothrombinase-inhibition induced by Bitis snake venoms. Comp. Biochem. Physiol. Part C Toxicol. Pharmacol..

[53] Wang Y., Zhang J., Zhang D., Xiao H., Xiong S., Huang C. (2018). Exploration of the inhibitory potential of varespladib for snakebite envenomation. Molecules.

[54] Liu C.C., Wu C.J., Hsiao Y.C., Yang Y.H., Liu K.L., Huang G.J., Hsieh C.H., Chen C.K., Liaw G.W. (2021). Snake venom proteome of *Protobothrops mucrosquamatus* in Taiwan: Delaying venom-induced lethality in a rodent model by inhibition of phospholipase A2 activity with varespladib. J. Proteom..

[55] Salvador G.H.M., Gomes A.A.S., Bryan-Quirós W., Fernández J., Lewin M.R., Gutiérrez J.M., Lomonte B., Fontes M.R.M. (2019). Structural basis for phospholipase A2-like toxin inhibition by the synthetic compound Varespladib (LY315920). Sci. Rep..

[56] Lewin M., Samuel S., Merkel J., Bickler P. (2016). Varespladib (LY315920) appears to be a potent, broad-spectrum, inhibitor of snake venom phospholipase A2 and a possible pre-referral treatment for envenomation. Toxins.

[57] Lewin M.R., Gilliam L.L., Gilliam J., Samuel S.P., Bulfone T.C., Bickler P.E., Gutiérrez J.M. (2018). Delayed LY333013 (oral) and LY315920 (intravenous) reverse severe neurotoxicity and rescue juvenile pigs from lethal doses of *Micrurus fulvius* (eastern coral snake) venom. Toxins.

[58] Lewin M.R., María Gutiérrez J., Samuel S.P., Herrera M., Bryan-Quirós W., Lomonte B., Bickler P.E., Bulfone T.C., Williams D.J. (2018). Delayed oral LY333013 rescues mice from highly neurotoxic, lethal doses of papuan taipan (*Oxyuranus scutellatus*) venom. Toxins.

[59] Zinenko O., Tovstukha I., Korniyenko Y. (2020). PLA2 inhibitor varespladib as an alternative to the antivenom treatment for bites from nikolsky’s viper *Vipera berus nikolskii*. Toxins.

[60] Gutiérrez J.M., Lewin M.R., Williams D.J., Lomonte B. (2020). Varespladib (LY315920) and methyl varespladib (LY333013) abrogate or delay lethality induced by presynaptically acting neurotoxic snake venoms. Toxins.

[61] Neumann C., Slagboom J., Somsen G.W., Vonk F., Casewell N.R., Cardoso C.L., Kool J. (2020). Development of a generic high-throughput screening assay for profiling snake venom protease activity after high-resolution chromatographic fractionation. Toxicon.

[62] Still K.B.M., Nandlal R.S.S., Slagboom J., Somsen G.W., Casewell N.R., Kool J. (2017). Multipurpose HTS coagulation analysis: Assay development and assessment of coagulopathic snake venoms. Toxins.

[63] Kazandjian T.D., Robinson S.D., Greene H.W., Carter D.A., Wouters R.M., Wagstaff S.C., Arias A.S., Albulescu L.-O., McCabe C.V., da Silva R.R. (2020). Convergent Evolution of Pain-Inducing Defensive Venom Components in Spitting Cobras. bioRxiv.

