# Supplementary material for: Anticoagulant Activity of Naja nigricollis Venom Is Mediated by Phospholipase A2 Toxins and Inhibited by Varespladib
Source: Toxins (Basel). 2021 Apr 23;13(5):302. doi: 10.3390/toxins13050302 (PMC8145175; doi:10.3390/toxins13050302)
Supplement: Supplementary file 1 [file toxins-13-00302-s001.zip › toxins-1159195-supplementary.pdf]

Supplementary materials: Anticoagulant Activity of *Naja nigricollis* Venom is Mediated by Phospholipase A2 Toxins and Inhibited by Varespladib

Taline Kazandjian, Arif Arrahman, Kristina B.M. Still, Govert W. Somsen, Freek J. Vonk, Nicholas R. Casewell, Mark C. Wilkinson and Jeroen Kool

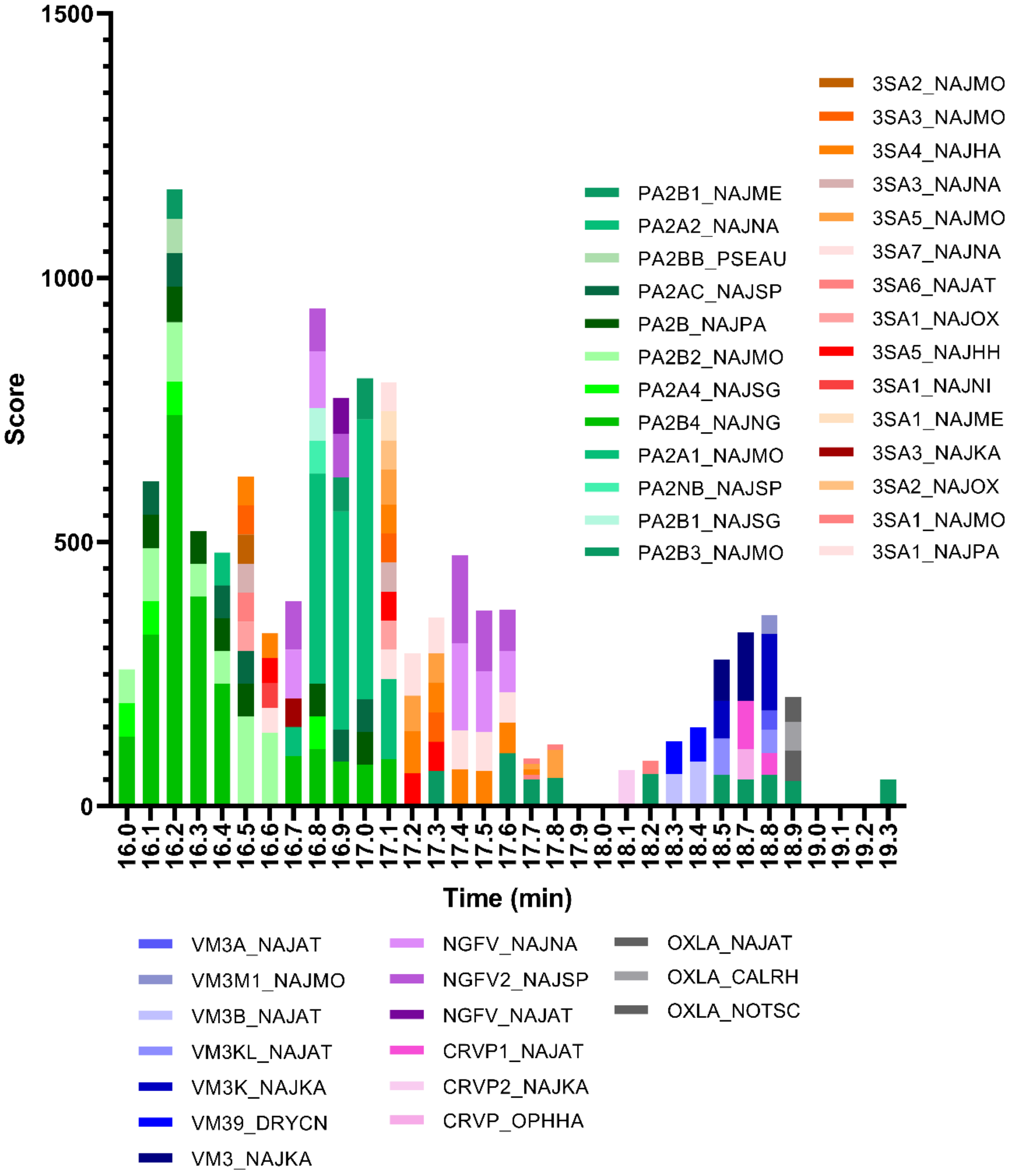

Figure S1. Detailed results of proteomics searches using Mascot software against the Swiss-Prot database to identify anti-coagulant venom proteins. The protein score represents the probability that designated proteins are present at the sample. PA stands for phospholipase A_2, 3S stands for 3FTxs, VM stands for snake venom metalloproteinases, NGFV stands for venom nerve growth factor, CRVP stands for cysteine-rich venom proteins, and OXLA stands for L-amino-acid oxidase.

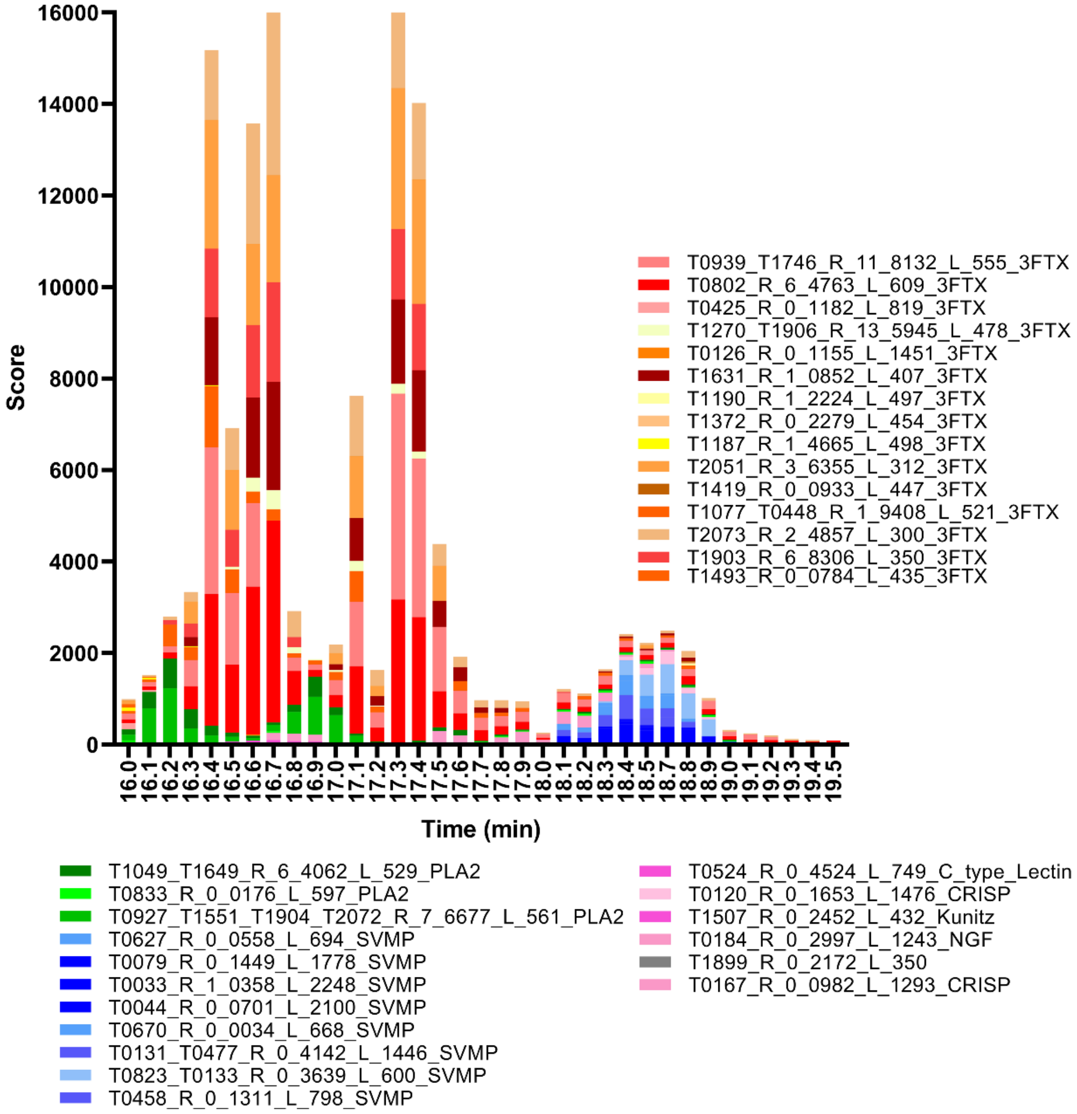

Figure S2. Detailed results of proteomics searches using Mascot software against the *N. nigricollis* species-specific venom gland transcriptome-derived database. The protein score represents the probability that designated protein is present at the sample.

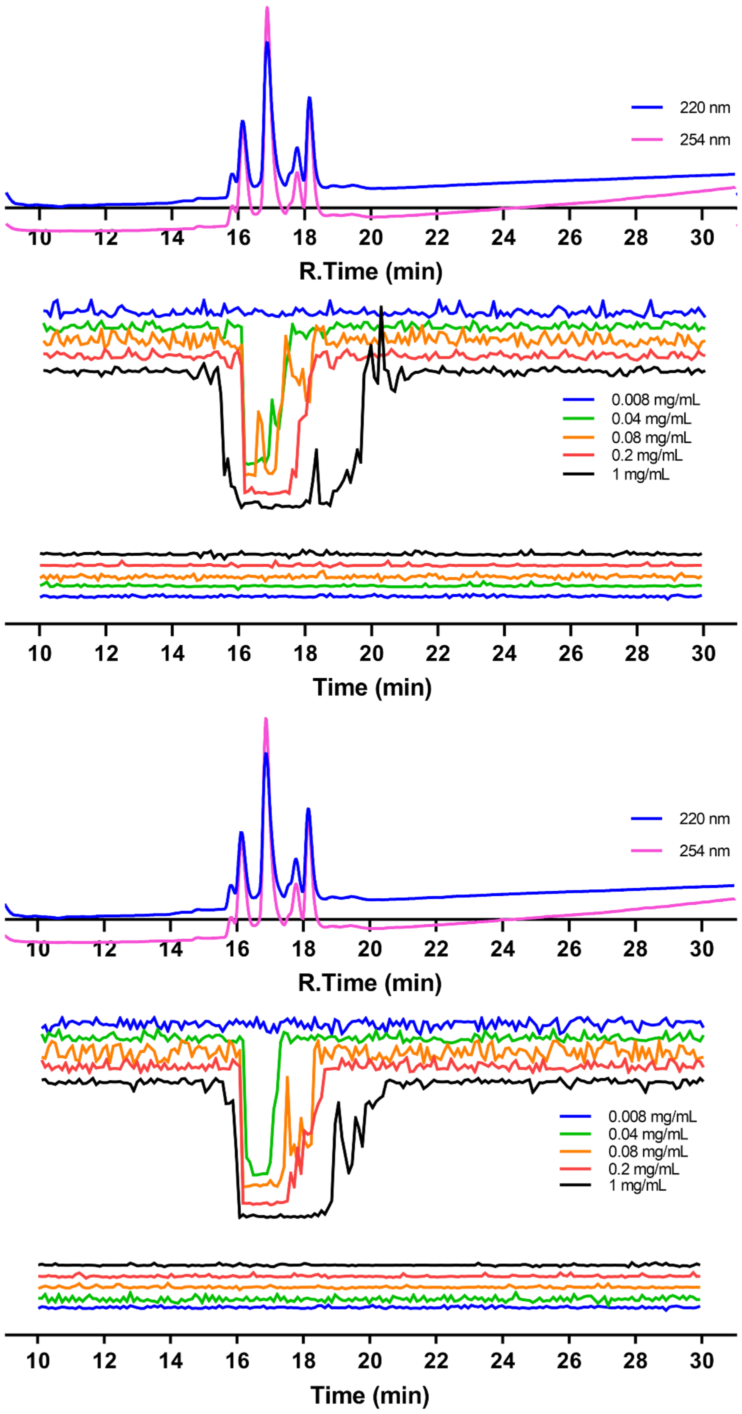

Figure S3. Duplication results of superimposed pro- and anticoagulation bioassay chromatograms of *N. nigricollis* venom. The duplicate data shown results from analyses of serial diluted *N. nigricollis* venom ranging from 1 mg/mL to 0.008 mg/mL (50 µL per injection).

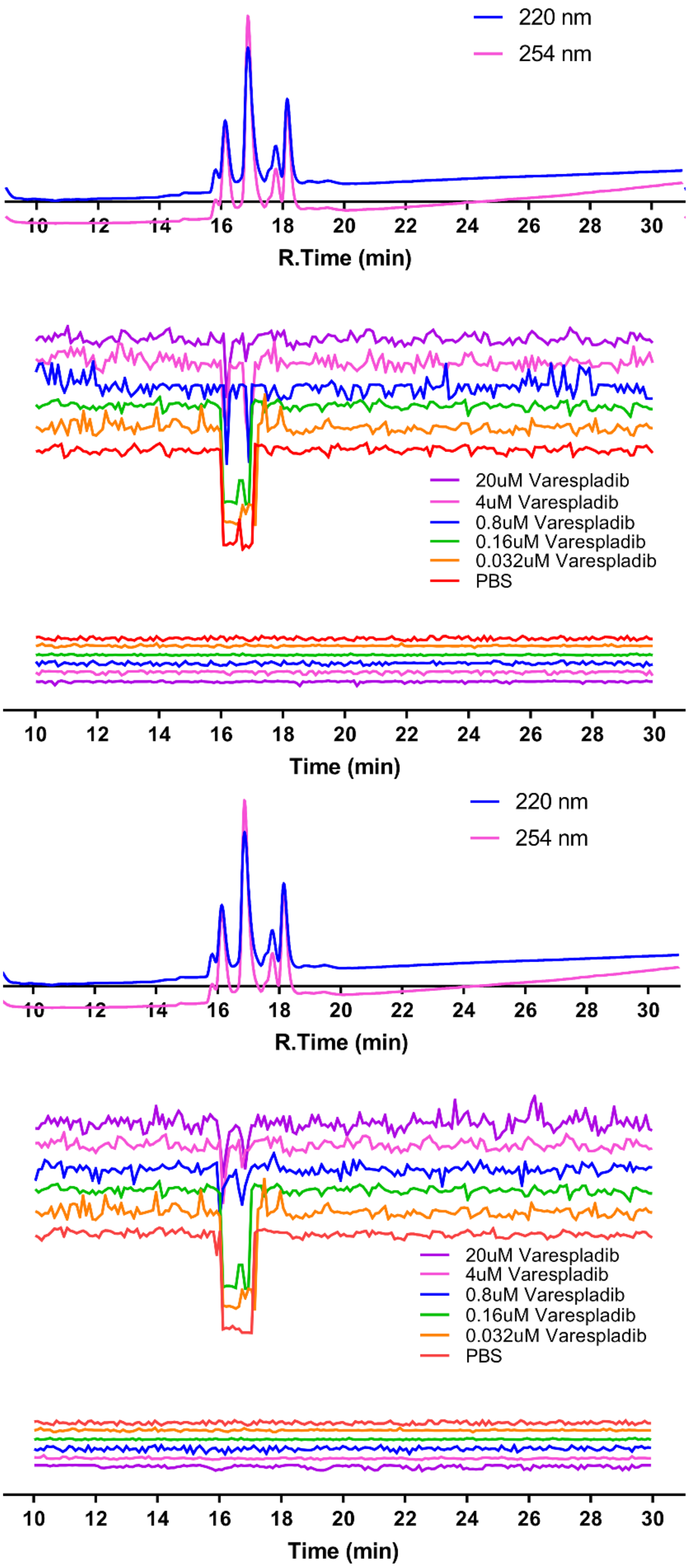

Figure S4. Duplication results of superimposed bioassay chromatograms resulting from analyses of *N. nigricollis* venom (0.2 mg/mL, 50 µL injection volume) in the presence of different concentrations of varespladib. Varespladib effectively inhibited anticoagulant venom effects in a dose dependent manner with inhibitor concentrations ranging from 20 µM to 0.032 µM.

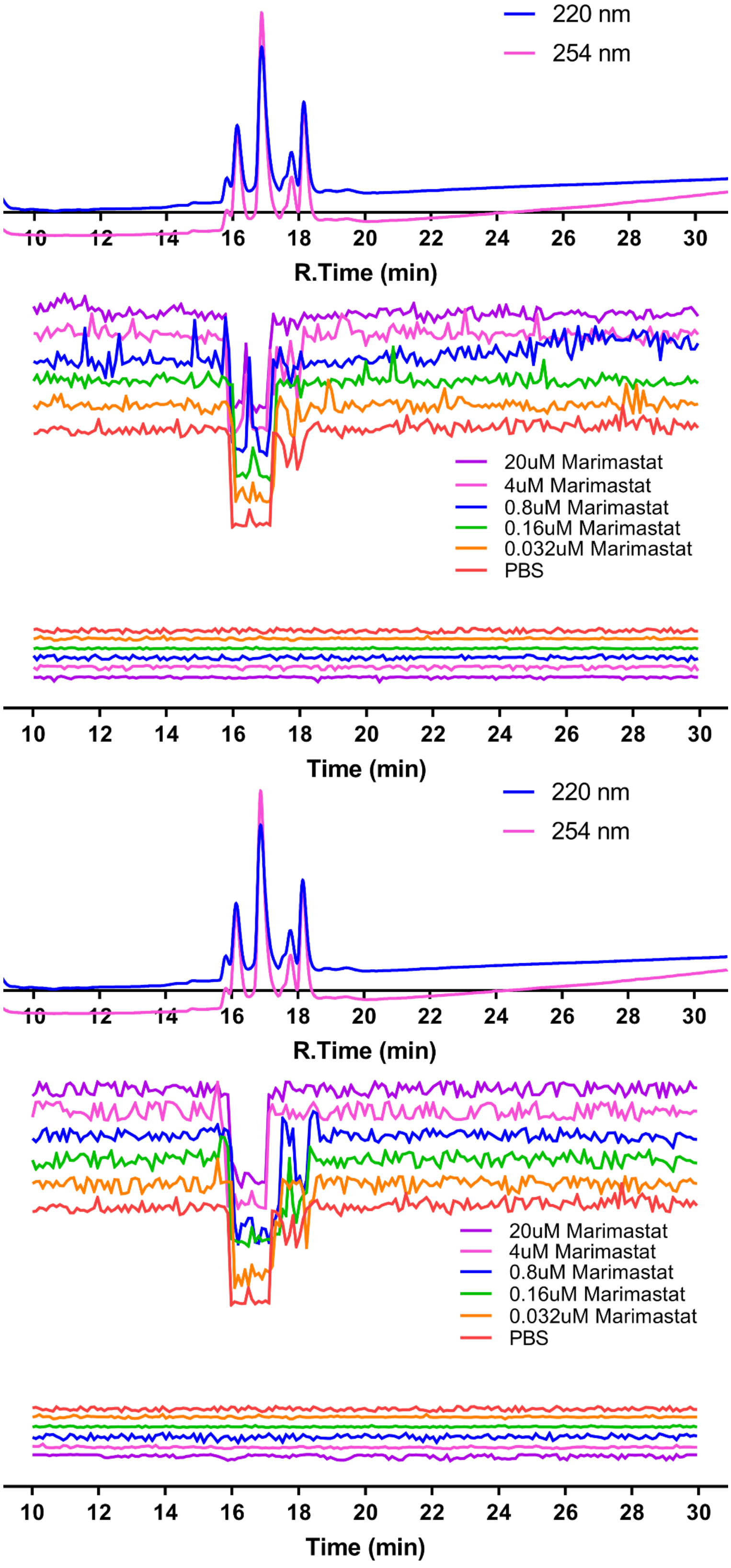

Figure S5. Duplication results of superimposed bioassay chromatograms resulting from analyses of *N. nigricollis* venom (0.2 mg/mL, 50 µL injection volume) in the presence of different concentrations of Marimastat. Marimastat had no inhibitory effect on anticoagulant activity. The inhibitor concentrations ranged from 20 µM to 0.032 µM.
